# Supplementary figures and images for: MALDI-MSI as a Complementary Diagnostic Tool in Cytopathology: A Pilot Study for the Characterization of Thyroid Nodules
Source: Cancers (Basel). 2019 Sep 16;11(9):1377. doi: 10.3390/cancers11091377 (PMC6769566; doi:10.3390/cancers11091377)

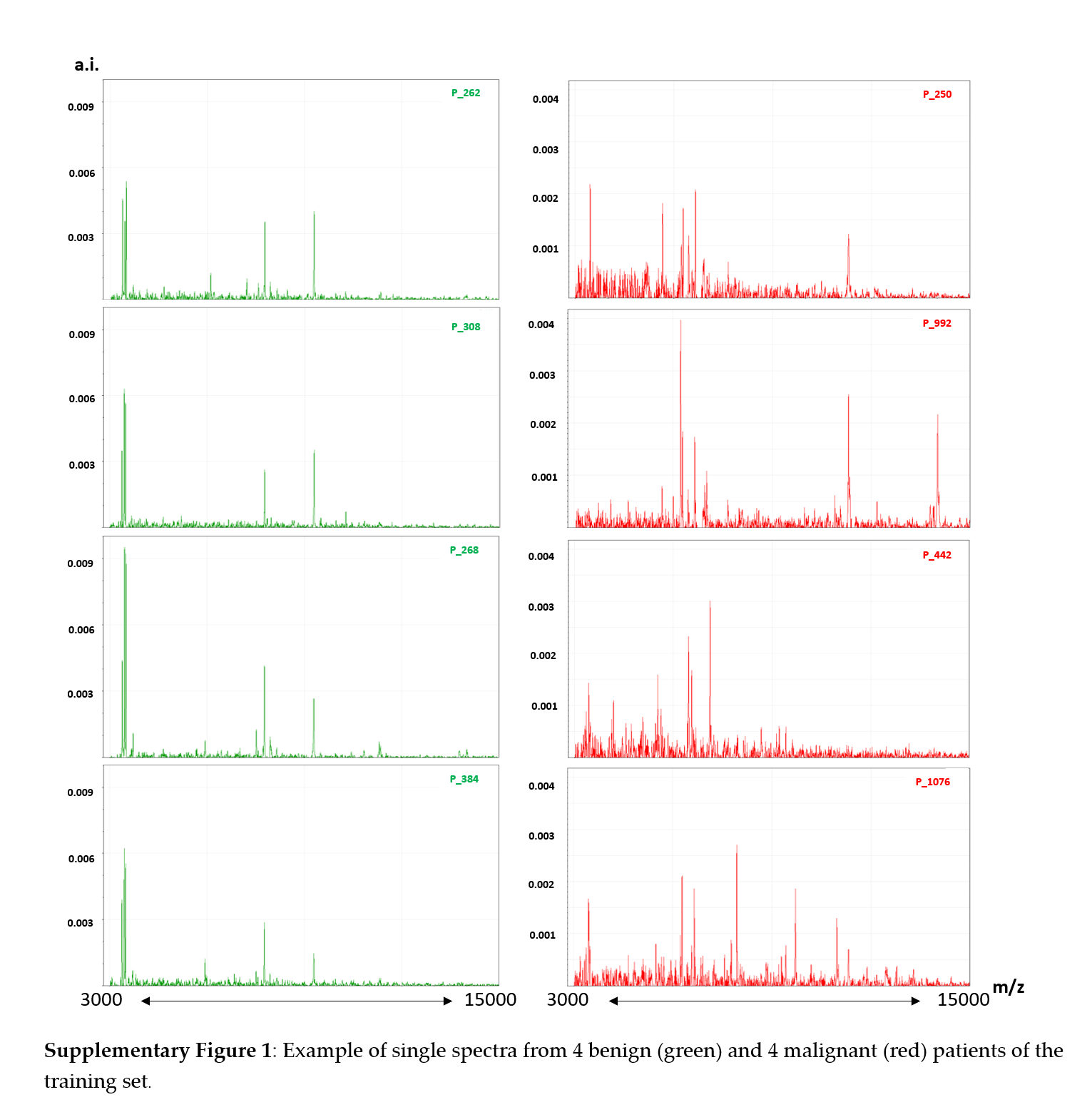

Supplement: Supplementary file 1 [file cancers-11-01377-s001.zip › supplementary materials/Supplementary Figure 1.tif]

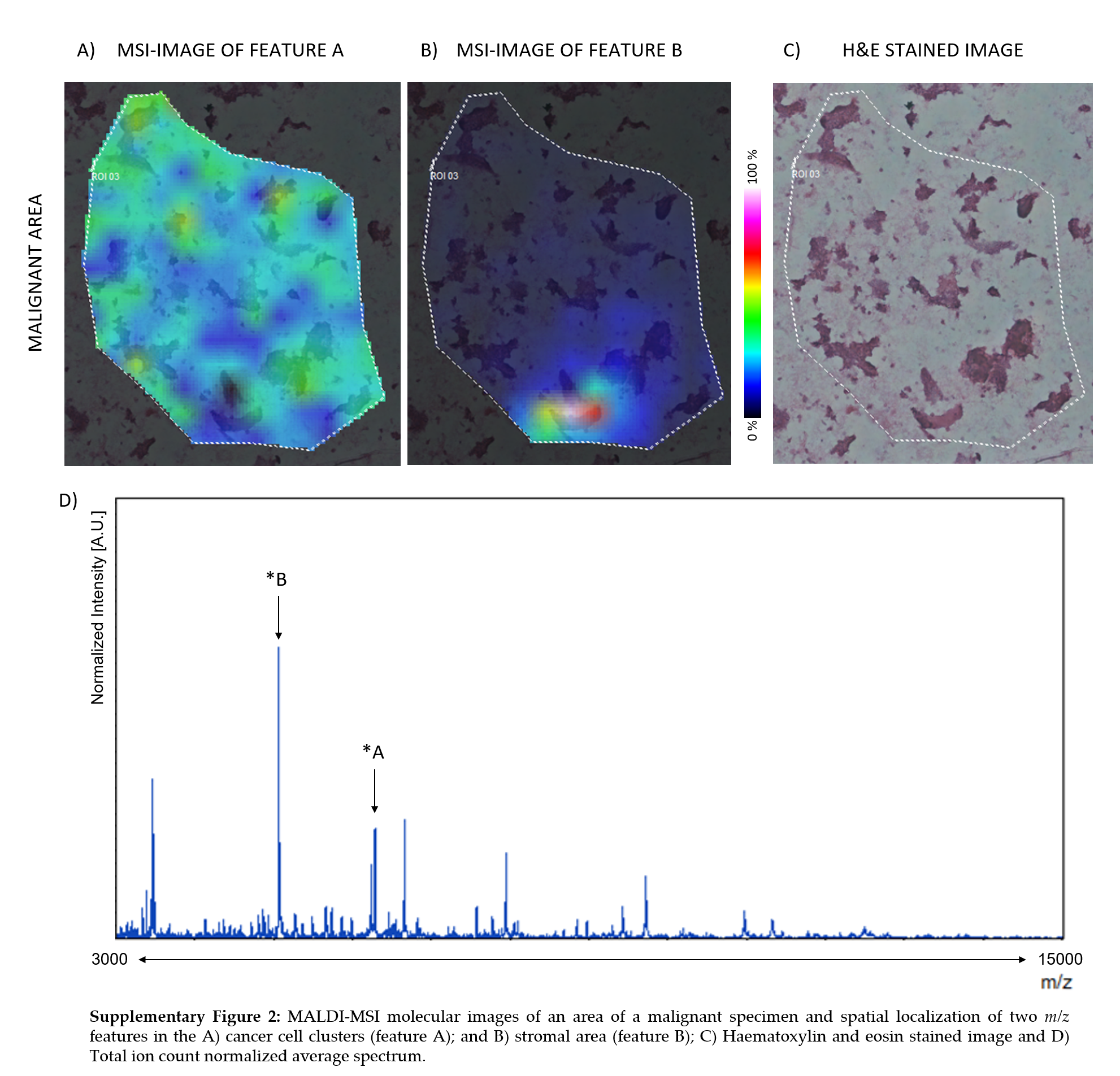

Supplement: Supplementary file 1 [file cancers-11-01377-s001.zip › supplementary materials/Supplementary Figure 2.tif]
